# Characterization of Mono- and Bi-Transgenic Pig-Derived Epidermal Keratinocytes Expressing Human *FUT2* and *GLA* Genes—In Vitro Studies

**DOI:** 10.3390/ijms22189683

**Published:** 2021-09-07

**Authors:** Jerzy Wiater, Marcin Samiec, Kamil Wartalski, Zdzisław Smorąg, Jacek Jura, Ryszard Słomski, Maria Skrzyszowska, Marek Romek

**Affiliations:** 1 Department of Histology, Jagiellonian University Medical College, Kopernika 7 Street, 31-034 Kraków, Poland; jerzy.wiater@uj.edu.pl (J.W.); kamil.wartalski@uj.edu.pl (K.W.); 2 Department of Reproductive Biotechnology and Cryoconservation, National Research Institute of Animal Production, Krakowska 1 Street, 32-083 Balice near Kraków, Poland; zdzislaw.smorag@iz.edu.pl (Z.S.); jacek.jura@iz.edu.pl (J.J.); maria.skrzyszowska@iz.edu.pl (M.S.); 3 Institute of Human Genetics, Polish Academy of Sciences, Strzeszyńska 32 Street, 60-479 Poznań, Poland; slomski@up.poznan.pl; 4 Department of Biochemistry and Biotechnology, Poznań University of Life Sciences, Dojazd 11 Street, 60-647 Poznań, Poland; 5 Department of Cell Biology and Imaging, Institute of Zoology and Biomedical Research, Jagiellonian University in Kraków, Gronostajowa 9 Street, 30-387 Kraków, Poland

**Keywords:** genetically modified pig, epidermal keratinocyte, human α-1,2-fucosyltransferase, human α-galactosidase A, Galα1→3Gal epitope

## Abstract

Pig-to-human xenotransplantation seems to be the response to the contemporary shortage of tissue/organ donors. Unfortunately, the phylogenetic distance between pig and human implies hyperacute xenograft rejection. In this study, we tested the hypothesis that combining expression of human α1,2-fucosyltransferase (h*FUT2*) and α-galactosidase A (h*GLA*) genes would allow for removal of this obstacle in porcine transgenic epidermal keratinocytes (PEKs). We sought to determine not only the expression profiles of recombinant human α1,2-fucosyltransferase (rhα1,2-FT) and α-galactosidase A (rhα-Gal A) proteins, but also the relative abundance (RA) of Galα1→3Gal epitopes in the PEKs stemming from not only h*FUT2* or h*GLA* single-transgenic and h*FUT2*×h*GLA* double-transgenic pigs. Our confocal microscopy and Western blotting analyses revealed that both rhα1,2-FT and rhα-Gal A enzymes were overabundantly expressed in respective transgenic PEK lines. Moreover, the semiquantitative levels of Galα1→3Gal epitope that were assessed by lectin fluorescence and lectin blotting were found to be significantly diminished in each variant of genetically modified PEK line as compared to those observed in the control nontransgenic PEKs. Notably, the bi-transgenic PEKs were characterized by significantly lessened (but still detectable) RAs of Galα1→3Gal epitopes as compared to those identified for both types of mono-transgenic PEK lines. Additionally, our current investigation showed that the coexpression of two protective transgenes gave rise to enhanced abrogation of Galα→3Gal epitopes in h*FUT2*×h*GLA* double-transgenic PEKs. To summarize, detailed estimation of semiquantitative profiles for human α-1,2-FT and α-Gal A proteins followed by identification of the extent of abrogating the abundance of Galα1→3Gal epitopes in the ex vivo expanded PEKs stemming from mono- and bi-transgenic pigs were found to be a sine qua non condition for efficiently ex situ protecting stable lines of skin-derived somatic cells inevitable in further studies. The latter is due to be focused on determining epigenomic reprogrammability of single- or double-transgenic cell nuclei inherited from adult cutaneous keratinocytes in porcine nuclear-transferred oocytes and corresponding cloned embryos. To our knowledge, this concept was shown to represent a completely new approach designed to generate and multiply genetically transformed pigs by somatic cell cloning for the needs of reconstructive medicine and dermoplasty-mediated tissue engineering of human integumentary system.

## 1. Introduction

Nowadays, the use of the porcine cells, tissues, and organs seems to be the response to the contemporary scarcity of organs for transplantation. Porcine organs display a vast variety of anatomo-histological and physiological similarities to their human counterparts. Furthermore, the extent of genetic identity between these two mammalian species oscillates at a level of approximately 96% [1,2,3,4,5]. Pigs are characterized by high rates of fertility and prolificacy, which reflects in both enhanced breeding potential and rapid gain of specimen-specific body mass in this livestock species displaying relatively low or negligible incidence of time- and cost-consuming production.

Unfortunately, the phylogenetic distance between pigs and humans implies complex immune response, leading to either hyperacute rejection (HAR) or acute humoral and cellular rejection of porcine xenografts [6,7,8]. The HAR is an immediate reaction of human immune system to the high frequency of occurrence noticed for Galα1→3Gal epitopes undergoing expression on the external surface of porcine cells’ plasmalemma compartments. The biogenesis of Galα1→3Gal antigenic determinants is enzymatically catalyzed by α1,3-galactosyltransferase (α1,3-GT), which is encoded by *GGTA1* gene. The α1,3-GT enzyme displays the capability to biocatalyse the biochemical reactions of transferring galactose moieties from uridine 5′-diphosphogalactose (UDP-Gal) residues followed by α1→3 glycosidic binding these monosaccharide molecules to glycoproteins or glycosphingolipids containing terminal Galβ1→4GlcNAc-R residues [9,10]. Therefore, genetically engineering triggering the simultaneous coexpression of recombinant human α1,2-fucosyltransferase (rhα1,2-FT) and α-galactosidase A (rhα-Gal A) enzymes, which are encoded by h*FUT2* and h*GLA* transgenes, appears to be a promising approach to overcome the HAR of porcine cell, tissue, and organ xenotransplants [11,12]. Both endogenous porcine α1,3-GT and the transgenically expressed rhα1,2-FT utilize *N*-acetyllactosamine (LacNAc) residues for the purposes of their biocatalytic activities. However, human α1,2-FT occurs in the *cis* compartment of the Golgi apparatus and acts earlier than porcine endogenous α1,3-GT, which is present in the *trans* compartment of this organelle. As oligosaccharide moves through the Golgi apparatus, it is primarily fucosylated by rhα1,2-FT, whereby it cannot accept the terminal galactose residue in the subsequent reaction biocatalysed by α1,3-GT. This strategy is based on the competition of these two enzymes acting on the same substrate during oligosaccharide processing within transgenic cells [12,13]. In turn, the rhα-Gal A enzyme is responsible for the cleavage of terminal *D*-galactose moieties [14]. Zeyland et al. [15] reported successfully generating and multiplying the double-transgenic pigs displaying robust and ubiquitous expression of enzymatic rhα1,2-FT and rhα-Gal A immunoproteins for the first time. These investigators also demonstrated considerably dwindling the incidence of α-Gal antigenic determinants on the extracellular surface of porcine cutaneous fibroblast’s plasma membranes. However, the effect of genetically engineered modification of the aforementioned immunoenzymes may vary among different cell types due to their specific glycosylation patterns. To our knowledge, no research has ever been conducted to comprehensively estimate not only the concomitant semiquantitative profiles (RAs) of enzymatic rhα1,2-FT and rhα-Gal A immunoproteins, but also enhanced abrogation of Galα1→3Gal epitopes in the extracorporeally proliferating PEK lines that were previously established from dermal explants of the h*FUT2*×h*GLA* bi-transgenic pigs.

The ex vivo expanding and cryogenically protecting mono- and bi-transgenic adult epidermal cells (keratinocytes) that were thoroughly characterized by recognizing proteomic signatures related to semiquantifying not only led to the overabundance of rhα1,2-FT and/or rhα-Gal A enzymes, but also augmented downregulation of Galα1→3Gal antigenic determinants, which seem to be indispensable to subsequently investigating the suitability of PEKs for producing genetically engineered cloned pigs by somatic cell nuclear transfer (SCNT). The use of somatic cell cloning for multiplication of genetically transformed pigs, whose skin (including all its principal layers such as epidermis, dermis, and hypodermis) displays robust and ubiquitous expression profiles of rhα1,2-FT and rhα-Gal A proteins and, concomitantly, diminished RA of Galα1→3Gal epitopes, appears to be reliable and feasible strategy required to design different models of porcine dermo-epidermal bioprostheses or substitutes of human skin. The latter are intended for minimally invasive procedures of surgically repairing damaged or injured human skin by xenotransplanting porcine cutaneous grafts. These procedures are inevitable not only in regenerative medicine and tissue engineering of human dermo-integumentary system, but also in dermoplasty based on reconstructive surgery, which is mediated by xenogeneic skin grafting. The aforementioned tools of modern plastic surgery and reconstructive medicine are focused on applying the porcine genetically engineered skin bioprostheses to replacement or removal of: (1) congenital malformations of human dermo-integumentary system; (2) dermodysplastic lesions triggered by postoperative (surgical) skin wounds and scars or skin burn wounds and scars or accidental and inflicted skin injuries; (3) precancerous (premalignant) skin lesions in patients afflicted with e.g., precancerous keratosis; (4) cancerous skin tumors (malignant neoplasms) designated as metastatic cancers; (5) noncancerous oncogenic skin alterations resulting in the formation of benign (nonmalignant) tumors; and (6) degenerative changes in aging skin.

Cumulatively, it is beyond any doubt that a variety of comprehensive approaches used to determine the proteomic profile of permanent and homogenous cell lines of PEKs, which were successfully established from primary cultures stemming from skin-derived bioptates of mono- and bi-transgenic pigs, were applied for the first time. These approaches were accomplished to ex situ cryopreserve the reservoirs providing epigenetically reprogrammable nuclear donor epidermal cells (NDECs) for future studies aimed at cloning multigenetically transformed pigs by SCNT. To the best of our knowledge, the conceptualization of utilizing porcine single- or double-transgenic cutaneous keratinocytes as a source of terminally differentiated NDECs for efforts undertaken to generate somatic cell-cloned embryos, fetuses, and progeny in mammals was not yet developed. So far, the only sources of nuclear donor cells derived from dermo-integumentary system of adult mammalian specimens that were used for creation of cloned embryos, conceptuses, and offspring in mice originate from multipotent hair follicle bulge-derived epithelial stem cells and partially differentiated nonbulge keratinocyte progenitor cells, i.e., transit amplifying cells isolated from the basal layer of the epidermis and the upper outer root sheath of the hair follicles [16]. Therefore, attempts, which are targeted at exploring the capabilities of genetically modified cell nuclei inherited from adult epidermal keratinocytes to be epigenetically reprogrammed in porcine SCNT-derived oocytes and resultant embryos, are found to be a completely new strategy elaborated for the purpose of somatic cell cloning of pigs and other mammalian species.

## 2. Results

### 2.1. Fluorescent Immunolocalization of Recombinant Human α1,2-Fucosyltransferase (rhα1,2-FT) and α-Galactosidase A (rhα-Gal A) Enzymes in the Ex Vivo-Expanded Single- and Double-Transgenic PEKs

**Figure 1:**
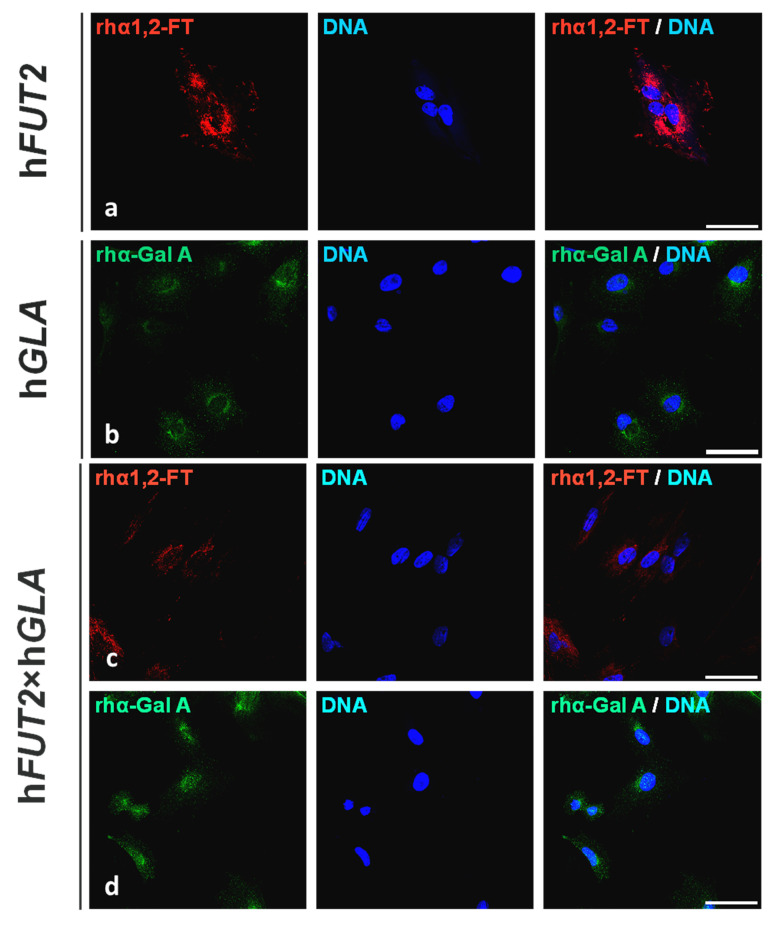
Immunofluorescence analysis of ex vivo-expanded PEKs originating from different variants of genetically engineered pigs detailed as: h*FUT2* mono-transgenic (**a**), h*GLA* mono-transgenic (**b**) and h*FUT2*×h*GLA* bi-transgenic (**c**,**d**). Representative microphotographs depicting immunofluorescent detection of rhα1,2-FT-related (**a**,**c**) and rhα-Gal A-related (**b**,**d**) locations recognized in single- and double-transgenic PEKs, respectively. Immunostaining with aid of red Cy3 fluorochrome-tagged or green Alexa Fluor 488 fluorochrome-tagged secondary antibodies and blue fluorescent nuclear counterdyeing with use of 4′,6-diamidino-2-phenylindole (DAPI). Scale bars represent 50 μm. Centers of immunofluorescently labelling the rhα1,2-FT enzymes were primarily localized in the perinuclear region of all analyzed cells (**a**,**c**). rhα-Gal A-descended signaling points were found to be homogeneously distributed in whole cytoplasm of diagnosed cells from each transgenic variant (**b**,**d**).

**Figure 2:**
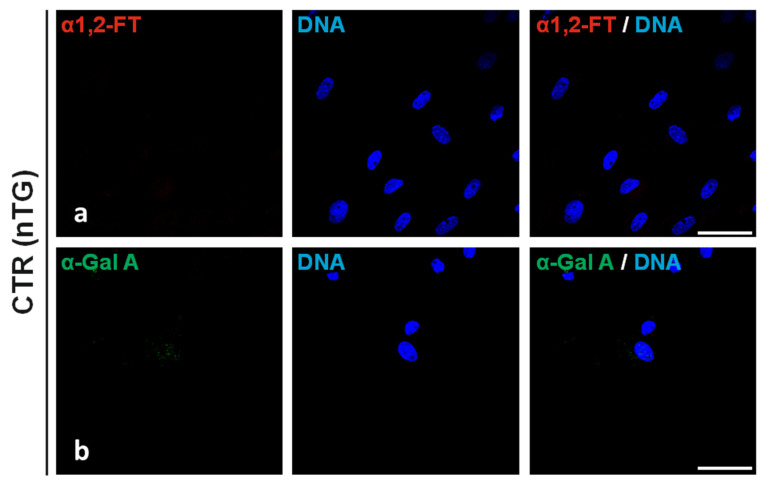
Immunofluorescence analysis of ex vivo-expanded PEKs stemming from control nontransgenic pigs (CTR nTG). Representative microphotographs depicting the immunofluorescent detection of α1,2-FT-related (**a**) and α-Gal A-related (**b**) locations identified in nontransgenic PEKs. Immunostaining with red Cy3 fluorochrome-tagged or green Alexa Fluor 488 fluorochrome-tagged secondary antibodies and blue fluorescent nuclear counterdyeing with the use of 4′,6-diamidino-2-phenylindole (DAPI). Scale bars represent 100 μm. No positive signaling points descended from either xenogeneic rhα1,2-FT or endogenous species-specific α1,2-FT were recognized (**a**), while sporadic incidence of hardly detectable spots correlated with immunofluorescently labelling the α-Gal A enzymes was noticed (**b**).

The regions of localization noticed for rhα1,2-FT and rhα-Gal A enzymes were determined by immunofluorescence staining of the in vitro cultured PEKs stemming from h*FUT2* or h*GLA* mono-transgenic (Figure 1), h*FUT2*×h*GLA* bi-transgenic (Figure 1), and nontransgenic (Figure 2) pigs served as a control group (CTR nTG). The positive rhα1,2-FT-derived immunofluorescence signal was predominantly confined to the perinuclear area in PEKs originating from h*FUT2* single-transgenic (Figure 1a) and h*FUT2*×h*GLA* double-transgenic (Figure 1c) cell lines. In turn, homogenously dispersed regions associated with fluorescently immunostaining the rhα-Gal A molecules were found to occur in whole cytoplasm of both transgenic PEK variants as follows: h*GLA* (Figure 1b) and h*FUT2*×h*GLA* (Figure 1d). But, in the porcine CTR nTG keratinocytes, we did not identify any positive signal descended from either extrinsic rhα1,2-FT or intrinsic species-specific α1,2-FT (Figure 2a), whereas the incidental locations related to immunofluorescently tagging the α-Gal A proteins were shown to be scarcely detectable (Figure 2b).

### 2.2. Western Blot-Mediated Determination of the Relative Expression Specific for Recombinant Human α1,2-Fucosyltransferase (rhα1,2-FT) and α-Galactosidase A (rhα-Gal A) Enzymes in the Ex Vivo-Expanded Single- and Double-Transgenic PEKs

**Figure 3:**
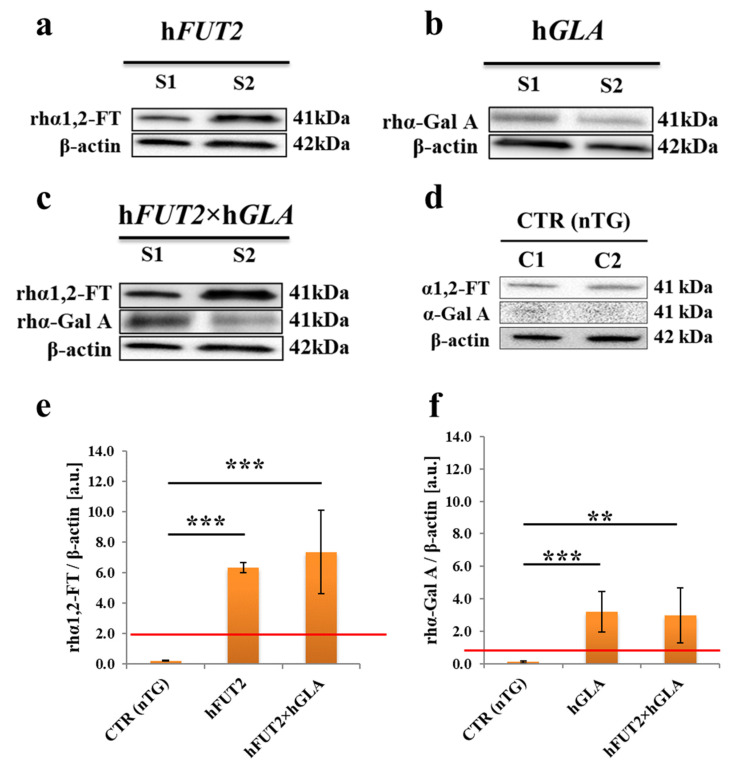
Western blot-mediated estimation of the semiquantitative profiles noticed for expression of recombinant human α1,2-fucosyltransferase (rhα1,2-FT) and α-galactosidase A (rhα-Gal A) enzymes in total protein samples extracted from the ex vivo-expanded control nontransgenic (CTR nTG) PEKs and their cell counterparts stemming from h*FUT2* and h*GLA* single-transgenic pigs and h*FUT2*×h*GLA* double-transgenic specimens. Representative blots corresponding to expression of rhα1,2-FT and rhα-Gal A enzymes in total protein samples that were isolated from PEKs stemming from h*FUT2* mono-transgenic (**a**), h*GLA* mono-transgenic (**b**), h*FUT2*×h*GLA* bi-transgenic (**c**) and CTR nTG pigs (**d**). β-Actin provides a loading control for all analyzed protein samples. Results of the semiquantitatively analyzing the relative abundances (RAs) determined for rhα1,2-FT and rhα-Gal A enzymes (in arbitrary units) were presented in panels (**e**,**f**), respectively. Bar graphs show mean ± standard error of mean (SEM) of relative optical density (ROD) descended from three separate analyses of three animals for each variant. Red line is taken as the cut-off value 1.0. Statistics: one-way analysis of variance (ANOVA) followed by Tukey’s honestly significant difference (HSD) post hoc test. Values that are denoted as ** and *** indicate incidence of statistically significant differences between experimental groups with a probability of occurring random errors at the levels of *p* < 0.01 and *p* < 0.001, respectively.

To carry out Western blot analysis, the total protein samples were isolated from the in vitro cultured PEKs stemming not only from h*FUT2* mono-transgenic, h*GLA* mono-transgenic, and h*FUT2*×h*GLA* bi-transgenic, but also from control nontransgenic (CTR nTG) pigs. Western blot analysis of total protein extracts descended from the ex vivo proliferating PEKs confirmed the occurrence of rhα1,2-FT and rhα-Gal A enzymes in all the corresponding transgenic samples (Figure 3a–c). In the CTR nTG group (Figure 3d), we identified a weak positive signal for intrinsic species-specific α1,2-FT and barely detectable signal for intrinsic α-Gal A. Pigs are characterized by the lack of α-Gal A protein expression, which stems from species-specific silencing both alleles of p*GLA* gene. Taking this into consideration, the epigenetic alterations resulting from the in vitro culture conditions seem to trigger, to a very limited extent, onset of transcriptional and translational activities for p*GLA* alleles and their transcribed mRNAs in the negligible pools of the ex vivo expanded porcine CTR nTG keratinocytes. Signal intensities of the analyzed proteins (rhα1,2-FT and rhα-Gal A) were normalized to β-actin, which provides a loading control. The semiquantitative analysis of Western blot clearly proved that the relative abundances (RAs) estimated for both tested enzymes were considered to be significantly augmented (at least *p* < 0.01) in the total protein samples from each transgenic variant of PEKs as compared to the RA determined for CTR nTG group (Figure 3e,f).

### 2.3. Lectin GS I-B_4_-Mediated Fluorocytochemically Detecting the Expression Profiles of Galα1→3 Gal Epitope in the Ex Vivo-Expanded Single-, Double- and Nontransgenic PEKs

**Figure 4:**
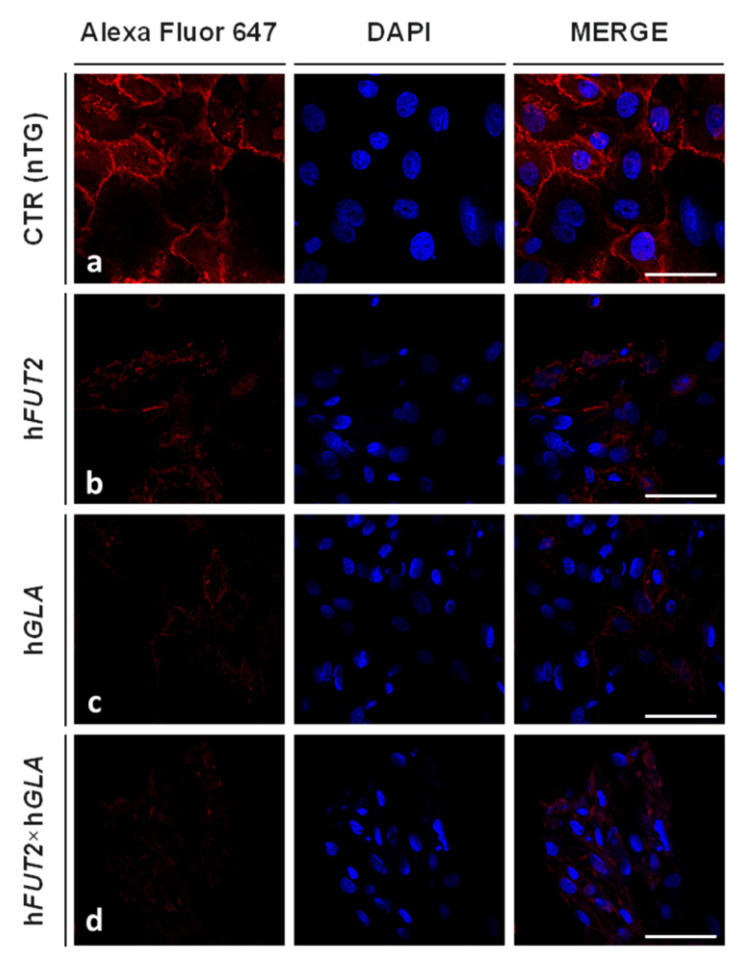
Lectin fluorescence analysis of expression profiles specific for Galα1→3Gal epitopes in ex vivo-expanded PEKs stemming either from genetically nontransformed pigs designated as CTR nTG group (**a**) or from different types of genetically transformed pigs as follows: h*FUT2* mono-transgenic (**b**), h*GLA* mono-transgenic (**c**) and h*FUT2*×h*GLA* bi-transgenic (**d**). Representative microphotographs depicting incidences of fluorocytochemically recognizing lectin GS I-B_4_-labelled Galα1→3Gal antigenic determinants localized in intracellular compartments and plasma membranes of PEKs from each experimental variant specified as: CTR nTG (**a**), h*FUT2* single-transgenic (**b**), h*GLA* single-transgenic (**c**), and h*FUT2*×h*GLA* double-transgenic (**d**). The lectin fluorescence analysis was accomplished by using Alexa Fluor 647-conjugated lectin GS I-B_4_ (highly specific red fluorocytochemical tagging of Galα1→3Gal antigenic determinants) and 4′,6-diamidino-2-phenylindole (DAPI) counterstain (blue dyeing of the cell nuclei). Scale bars represent 100 μm.

The expression levels noticed for Galα1→3Gal antigenic determinants were identified in the in vitro cultured PEKs by their tagging with Alexa Fluor 647-conjugated lectin GS I-B_4_. Figure 4 depicts the panels of microphotographs, in which the occurrence of Galα1→3Gal epitopes was fluorocytochemically confirmed in extracorporeally proliferating PEKs derived from control nontransgenic (CTR nTG; Figure 4a), h*FUT2* or h*GLA* mono-transgenic (Figure 4b,c, respectively) and h*FUT2*×h*GLA* bi-transgenic pigs (Figure 4d). The Alexa Fluor 647/lectin GS I-B_4_ conjugates strongly labelled Galα1→3Gal antigenic determinants in the intracellular compartments, cytoskeleton, membrane skeleton and plasmalemma of the PEKs stemming from the CTR nTG group (Figure 4a). In contrast, the PEKs that originated from not only h*FUT2* and h*GLA* mono-transgenic pigs (Figure 4b,c, respectively) but also h*FUT2*×h*GLA* bi-transgenic pigs (Figure 4d) were characterized by the incidences of lectin GS I-B_4_-assisted fluorocytochemically recognizing the Galα1→3Gal epitopes that were shown to be remarkably lower than that observed in the CTR nTG group.

**Figure 5:**
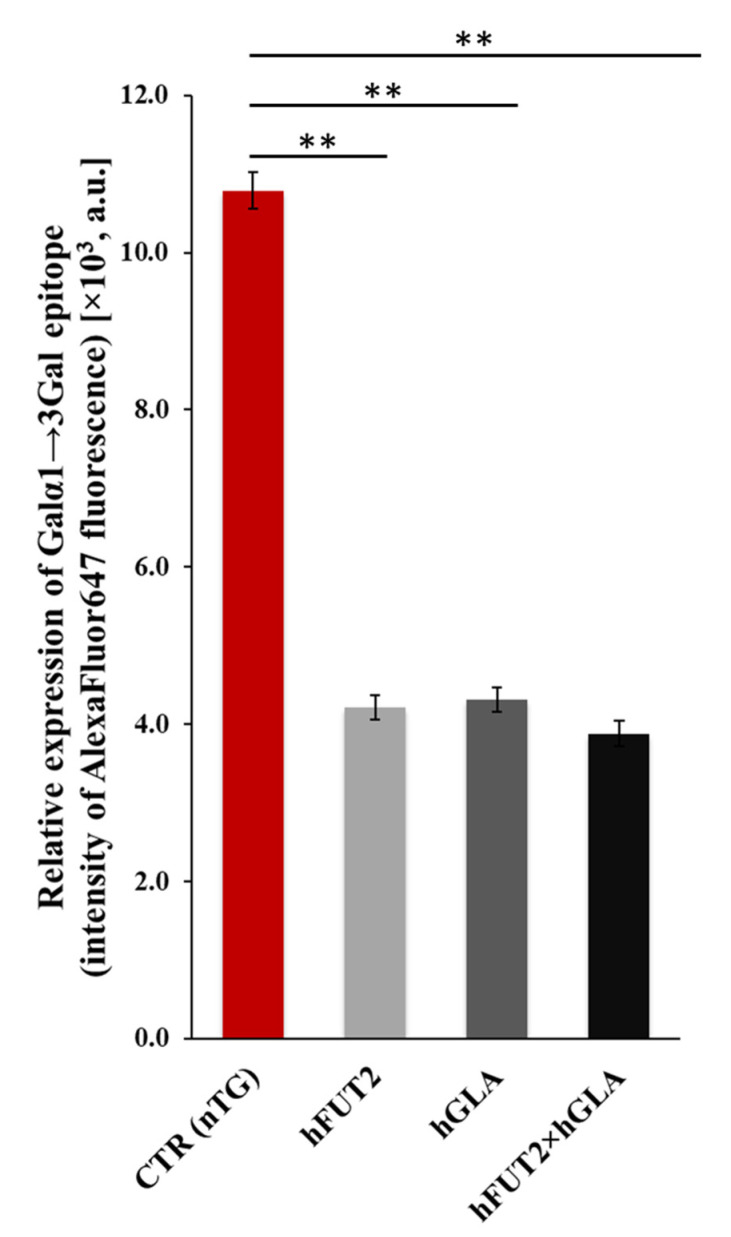
Semiquantitative analysis of fluorescence intensity of Alexa Fluor 647 dye for detecting expression of lectin GS I-B_4_-tagged Galα1→3Gal epitopes in the ex vivo-expanded PEKs stemming from control nontransgenic (CTR nTG) pigs and all the three types of transgenic specimens (h*FUT2,* h*GLA* and h*FUT2*×h*GLA*). For each variant, at least 70 regions of interest (ROI) of each field of view (*n* = 9) obtained from 5 animals were measured. Results on graph are presented in arbitrary units in form of exponential notation (×10^3^). Bars show mean ± standard error of the mean (SEM). Statistics: one-way analysis of variance (ANOVA) followed by Tukey’s honestly significant difference (HSD) post hoc test. Values that are denoted as ** indicate occurrence of statistically significant differences between experimental groups (*p* < 0.01). Relative expression of Galα1→3Gal epitope in all the tested cell samples (from each transgenic variant) were found to dwindle significantly as compared to that of CTR nTG group. PEKs derived from h*FUT2*×h*GLA* bi-transgenic pigs exhibited lowest expression of Galα1→3Gal antigenic determinants. Nonetheless, this expression level did not vary significantly from those identified in the h*FUT2* and h*GLA* mono-transgenic variants of PEKs (*p* ≥ 0.05).

Semi-quantitatively analyzing the fluorescence intensity (FI) of Alexa Fluor 647 dye (Figure 5) revealed that the expression levels estimated for lectin GS I-B_4_-tagged Galα1→3Gal epitopes were found to significantly wane in the PEKs of each transgenic variant as compared to their CTR nTG cell counterparts (*p* < 0.01). However, intergroup variability in the extents of Alexa Fluor 647-descended FI was not statistically proven among different types of genetically engineered PEK lines (*p* ≥ 0.05).

### 2.4. Lectin Blotting Analysis of Galα1→3Gal Epitope Expression at the Protein Level in the Ex Vivo-Expanded Single-, Double- and Nontransgenic PEKs

**Figure 6:**
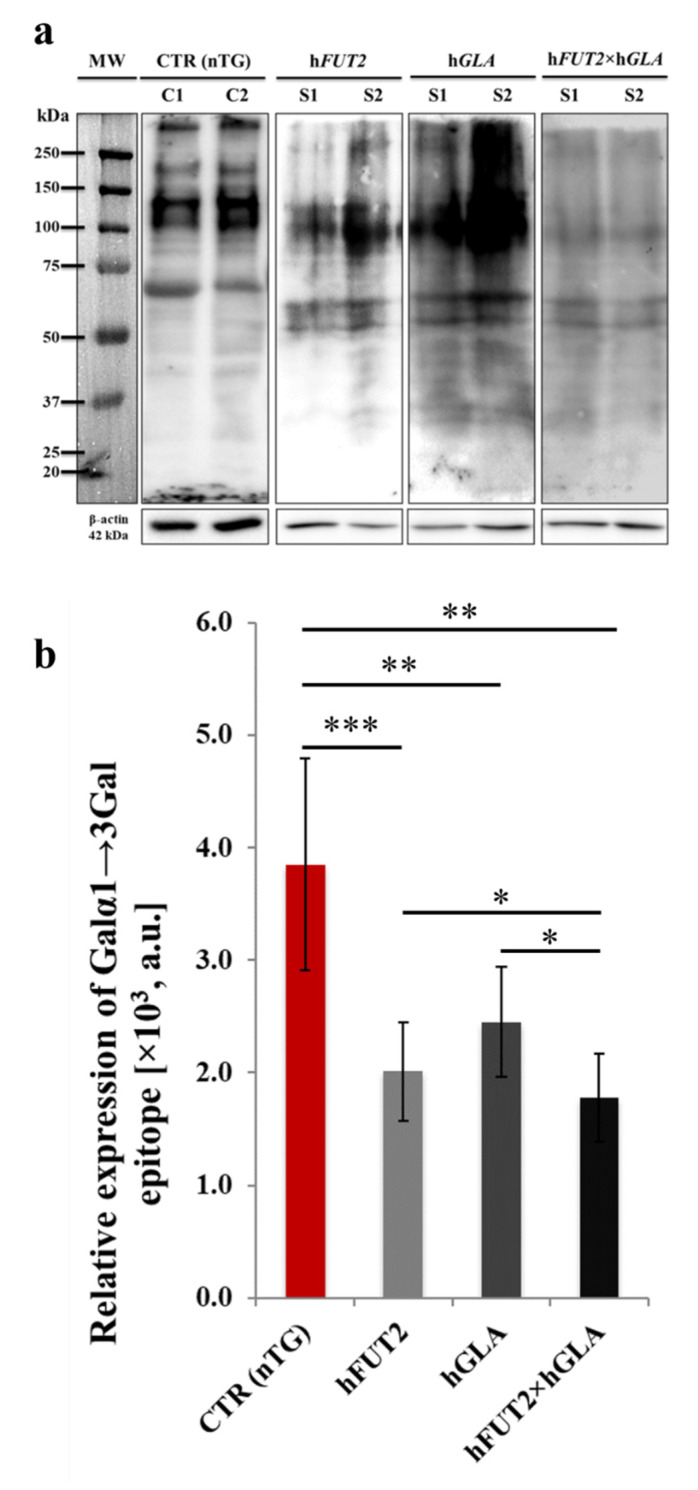
Analyzing semiquantitative profiles of Galα1→3Gal epitope expression by lectin blotting of protein samples isolated from ex vivo-expanded control nontransgenic (CTR nTG) PEKs and their cell counterparts stemming from h*FUT2* and h*GLA* single-transgenic pigs and h*FUT2*×h*GLA* double-transgenic specimens. (**a**): representative lectin blots corresponding to expression of Galα1→3Gal antigenic determinants in total protein samples that were extracted from PEKs originating from CTR nTG, mono- (h*FUT2*, h*GLA*) and bi-transgenic (h*FUT2*×h*GLA*) pigs. MW indicates molecular weight of protein standards. Control 1, control 2, sample 1, and sample 2 were designated as C1, C2, S1, and S2, respectively. Each band represents a glycosylated protein containing the Galα1→3Gal epitope. β-Actin served as a loading control for all analyzed protein samples. (**b**): semiquantitatively analyzing the relative abundances (RAs) estimated for Galα1→3Gal epitopes (in arbitrary units). Relative optical density (ROD) from three separate analyses of at least three animals for each variant is expressed as mean. Graph bar shows mean ± standard error of the mean (SEM). Statistics: one-way analysis of variance (ANOVA) followed by Tukey’s honestly significant difference (HSD) post hoc test. Values that are denoted as *, ** and *** indicate incidence of statistically significant differences between experimental groups with a probability of occurring random errors at levels of *p* < 0.05, *p* < 0.01, and *p* < 0.001, respectively. Relative expression recognized for Galα1→3Gal antigenic determinants was proven to be perpetuated at significantly diminished extents in all types/models of transgenic PEKs (h*FUT2*, h*GLA* and h*FUT2*×h*GLA*) as compared to that of the CTR nTG group. Definitely lowest RA of Galα1→3Gal epitopes was determined for total protein samples derived from h*FUT2*×h*GLA* bi-transgenic keratinocytes. This RA was also considered to recede significantly in relation to the protein samples descended from h*FUT2* and h*GLA* mono-transgenic PEKs, which have turned out to vary only unremarkably from each other.

By using horseradish peroxidase (HRP)-conjugated isolectin GS I-B_4_, lectin blot analysis was performed to estimate the relative abundance (RA) of Galα1→3Gal antigenic determinants at the total protein level. The occurrence of Galα1→3Gal epitopes in all analyzed protein samples, including those isolated from control nontransgenic (CTR nTG) PEKs and their cell counterparts originating from h*FUT2* and h*GLA* mono-transgenic pigs and h*FUT2*×h*GLA* bi-transgenic specimens (Figure 6a). In turn, β-Actin provides a loading control. The semiquantitative analysis of RAs determined for Galα1→3Gal epitope revealed the presence of statistically significant intergroup variability between the samples descended from CTR nTG keratinocytes and their equivalents derived from all types of genetically modified (h*FUT2*, h*GLA* and h*FUT2*×h*GLA*) PEKs (Figure 6b). The semiquantitative profile noticed for the expression of Galα1→3Gal antigenic determinants was shown to be reduced, to the largest extent, in the h*FUT2*×h*GLA* double-transgenic PEKs as compared to that of the CTR nTG group (*p* < 0.01; Figure 6b). However, the RA of Galα1→3Gal epitope observed for the h*FUT2*×h*GLA* bi-transgenic cells was found to be significantly lower than those identified in both h*FUT2* and h*GLA* mono-transgenic keratinocytes (*p* < 0.05; Figure 6b).

## 3. Discussion

Genetically modified pigs could become, in the near future, a tremendously valuable source of xenogeneic cells, tissues, or organs for transplantation into humans. In compliance with the above-mentioned finding, the efforts attempted to apply the PEK-mediated xenografting could provide research highlight and pivotal complement to the preclinical and clinical treatments that are targeted at cell/tissue engineering, regenerative medicine, and reconstructive surgery of skin defects caused by a variety of factors. At the present stage, the strategies undertaken to use the human keratinocyte-based cutaneous bioprostheses for personalized medicine are of a great importance, which is reflected in expanding the scale and scope of clinical significance for different dermoplasty-related therapies. Cell cultures of oral and epidermal keratinocytes have gained high popularity in surgical treatments of maxillofacial and oral cavities. The latter result especially from the loss of epithelial structures due to chemical or thermal burns, trauma, penetrating injuries in gunshot wounds, or various procedures of tumor ablation in the maxillofacial area [17,18,19,20].

In our current study, we focused on the effects of overexpression of rhα1,2-FT and rhα-Gal A proteins on the levels of Galα1→3Gal epitopes in the extracorporeally expanded PEKs derived from mono- and bi-transgenic pigs. Our lectinfluorescence and lectinblotting analyses revealed the considerable diminishments in the RAs noticed for Galα1→3Gal antigenic determinants in the PEK lines of each transgenic variant (h*FUT2*, h*GLA* and h*FUT2*×h*GLA*) as compared to that of ex vivo expanded PEKs stemming from nontransgenic pigs. Moreover, taking into consideration the double-transgenic PEKs, the semiquantitative profiles of Galα1→3Gal epitopes dwindled remarkably in relation to both their single-transgenic cell counterparts. Therefore, the results of this investigation clearly indicated that constitutive coexpression of cooperating enzymes such as rhα1,2-FT and rhα-Gal A turns out to be more efficient in biocatalytic removal of the Galα1→3Gal antigenic determinants from PEK plasma membranes than separate transgenically induced functional activities of either rhα1,2-FT or rhα-Gal A enzymatic proteins translated from single h*FUT2* or h*GLA* mRNA transcripts. Furthermore, these results are consistent with our previous research focused on the liver tissues of genetically engineered pigs displaying the same genotypes [21]. Our data also support beyond any doubt emerging findings and a strong evidence that multiple-transgenic pig models are required to successfully eliminate the major xenoantigen to prolong the survival of cells, tissues and organs after pig-to-human transplantation [22,23,24,25,26,27,28,29,30]. Further, mono-transgenic pigs, which provided a source of dermal explants for efficiently establishing PEK lines in our current study, were previously generated by intrapronuclear microinjection of porcine in vivo-fertilized zygotes with linearized gene constructs. The latter were composed of porcine cytomegalovirus (*CMV*) promoters and h*FUT2* or h*GLA* exon sequences [15,22,31]. In turn, h*FUT2*×h*GLA* bi-transgenic pigs were produced by subsequently interbreeding the h*FUT2* and h*GLA* mono-transgenic specimens. For those reasons, there is a risk that the expression levels of the introduced genes can tend to be decreased with the next generations of genetically engineered pigs, until they will were completely silenced. These phenomena were proven to occur frequently in the progeny derived from transgenic animals [22,31,32]. Kong et al. [33] showed that transgene expression is strongly related to gene copy number and methylation capacity in transgenic pigs. Moreover, random integration of a single gene construct or multiple gene constructs on the chromosomes also bring about suppressing the transcriptional activities of transgenes [34,35,36]. In turn, Folger et al. [37] found that foreign DNA that was randomly introduced into the nuclear genome leads to tandem integration of multiple transgene copies, most often designated as the so-called concatamers or series of linked transgene molecules. Such linkage results from homologous recombination between exogenous DNA molecules. But, more importantly, Kong et al. [33] also demonstrated that the number of integrated gene copies may vary depending on the type of tissue tested. This directly suggests that the use of intrapronuclear microinjection of gene constructs into the zygotes does not allow to accurately determine the number of gene copies inserted/incorporated into the genome of a given animal. Also, a slight increase in the copy number of the introduced gene due to homologous recombination between the transgene molecules in tandem repeats cannot be excluded [34,35,36]. Thus, it seems to be indispensable to check the gene copy number at each stage of generating transgenic animals. Zeyland et al. [15,31] and Lipiński et al. [22] confirmed that, taking into account the h*FUT2* transgene, the average integration was maintained at the level of 3 gene copies, while, considering the h*GLA* transgene, it was perpetuated at the level of 16 copies, both for mono- and bi-transgenic pigs. However, the above-mentioned studies were performed only on ear skin-derived fibroblasts originating from transgenic piglets and encompassed only DNA and/or RNA analyzes with the aid of RT-PCR and/or Southern blot hybridization. The present research was targeted at analyzing the expression of protein products for mRNA molecules transcribed from h*FUT2* and h*GLA* genes in the in vitro cultured PEKs stemming both from h*FUT2* or h*GLA* mono-transgenic pigs and from h*FUT2*×h*GLA* bi-transgenic pigs. Nonetheless, the immunofluorescence analyses that were accomplished with the use of antibodies against human α1,2-FT and α-Gal A proved the presence of these enzymes in PEKs derived from all the types/models of transgenic pigs. The signal was clear and specific for each of the analyzed transgenic variants. Western blot analysis supported the observations from the immunofluorescence reaction. A strongly positive signal in the form of specific bands was observed for each of the tested immunoproteins in the samples originating from all transgenic variants. On the other hand, semiquantitative analysis enabled to finally confirm the expression of the diagnosed proteins in the samples stemming from all the types of transgenic PEKs as compared to that of the CTR nTG group. Notably, slight (statistically nonsignificant) differences in the RAs of rhα1,2-FT and rhα-Gal were identified in keratinocytes derived from single-transgenic pigs (h*FUT2*, h*GLA*) and their bi-transgenic (h*FUT2*×h*GLA*) counterparts.

The panel of the current studies aimed at estimating the expression of the Galα1→3Gal epitopes with the use of fluorescently labelled isolectin GS I-B_4_ did not show unequivocally an additive effect of both enzymes (rhα1,2-FT and rhα-Gal) in porcine keratinocytes. In spite of the important fact that levels estimated for the relative expression of Galα1→3Gal antigenic determinants pivotally dropped in h*FUT2*×h*GLA* double-transgenic PEKs as compared to that identified in their nontransgenic cell counterparts, the inconsiderably declined expression profiles of these epitopes were recognized for bi-transgenic keratinocytes (fold change oscillating at the level of 2.78) in relation to their h*FUT2* and h*GLA* mono-transgenic cell equivalents. The additive effect of rhα1,2-FT and rhα-Gal A proteins was demonstrated only by the Eastern blot method using HRP-labelled isolectin GS I-B_4_. Houndebine et al. [34] and Zeyland et al. [15] also did not observe a clear synergistic effect of both enzymes. In our present investigation, the lack of a clearly additive effect of rhα1,2-FT and rhα-Gal A proteins may be because the analyzed keratinocytes stemmed from heterozygous pigs. The obtained results are consistent with the study by Zeyland et al. [15], in which researchers also sought to examine the pigs heterozygous for h*FUT2* and h*GLA* genes. The aforementioned researchers concluded that investigations targeted at generating and multiplying homozygous pigs are inevitable to verify the cumulative effect of these genes. Nevertheless, the results achieved in our current study and the literature data have justified the requirement of the use of pigs expressing both the rhα1,2-FT and rhα-Gal A, which seems to be the most promising solution for the experimental, preclinical, and clinical strategies attempted to carry out surgical treatments targeted at pig-to-human transplanting cell, tissue, or organ xenografts. The present investigation is another step towards recognizing mechanisms that underlie the phylogenetic (interspecies) immune barrier between pigs and humans. Finally, the achievements resulting from the current study and accessible literature data proved that a variety of approaches to genetic engineering are indispensable to be linked/combined each other to generate poly-transgenic pigs exhibiting stable incorporation of xenogeneic/heterologous genes into their nuclear genomes. This is a sine qua non condition for overcoming taxonomic (pig-to-human) hindrance to efficient surgical treatments encompassing xenotransplantation of cells, tissues, or organs.

## 4. Materials and Methods

### 4.1. Postmortem Collection of Skin Tissue Explants from Single-, Double- and Nontransgenic Pigs

All analyses were conducted on porcine dermal tissue bioptates that were postmortem recovered from not only h*FUT2* and h*GLA* mono-genetically engineered specimens but also their h*FUT2*×h*GLA* bi-transgenic and nontransgenic counterparts. All sampling and material procedures were carried out according to the protocols thoroughly described in our previous study [21]. In our current investigation, a total of 20 skin tissue samples derived from 10 mono-transgenic pigs (h*FUT2*, *n* = 5; h*GLA*, *n* = 5), 5 bi-transgenic pigs (h*FUT2*×h*GLA*, *n* = 5) and 5 nontransgenic pigs of Polish Large White breed (served as a control group) were utilized. Genetically transformed pigs were designed to display the robust and ubiquitous expression of either such recombinant human immuno-enzymes as: only rhα1,2-FT or rhα-Gal A (mono-transgenic animal models) or both rhα1,2-FT and rhα-Gal A (bi-transgenic animal models) [15,22]. Dermal tissue explants were retrieved from 12- to 18-month-old (i.e., postpubertal) animals at a body weight ranging from 150 to 200 kg. All animal procedures that were used in the studies by Zeyland et al. [15] and Wiater et al. [21] were performed in accordance with the European Directive 2010/63/EU and approved by the Second Local Ethics Committee in Kraków, Poland (Permission 1181/2015 from 21 May 2015). Collected dermal tissue bioptates were deposited into ice-cold transporting buffer, which was comprised of Ca^2+^- and Mg^2+^-free phosphate-buffered saline (PBS) solution (pH = 7.4; Biomed, Lublin, Poland) and supplemented with 3% (*v*/*v*) penicilin/streptomycin cocktail (Gibco^TM^; Thermo Fisher Scientific, Waltham, MA, USA) and 0.25 µg/mL amphotericin B (Gibco^TM^; Thermo Fisher Scientific). Antibiotically and antimycotically protected skin tissue samples were subsequently transported on wet ice into laboratory within 4 h.

### 4.2. Establishment of the Ex Vivo-Expanded Epidermal Keratinocytes Stemming from Skin Tissue Samples of Single-, Double- and Nontransgenic Pigs

The dermal tissue explants were rinsed thrice in sterile calcium/magnesium cation-deprived PBS (pH = 7.4; Biomed) supplemented with 3% (*v*/*v*) penicilin/streptomycin cocktail (Gibco^TM^; Thermo Fisher Scientific, Waltham, MA, USA) and 0.25 µg/mL amphotericin B (Gibco^TM^; Thermo Fisher Scientific). Afterwards, dermal tissue samples were carefully dissected and minced followed by 2-h enzymatic digestion of epidermal pieces with the aid of sterile 0.2% collagenase (Sigma–Aldrich; St. Louis, MO, USA) in PBS (Biomed) at a temperature of +37 °C. The cell suspensions were subsequently filtered through a 70-µm nylon cell strainers and washed in PBS (Biomed), followed by their triple centrifugation at 200× *g* for 5 min. In the next step, porcine epidermal keratinocytes (PEKs) were subcultured in Dulbecco’s Modified Eagle’s Medium/Nutrient Ham’s Mixture F-12 (DMEM/F-12) (1:1) (Gibco^TM^; Thermo Fisher Scientific) enriched with 5% fetal bovine serum (FBS; Gibco^TM^, Thermo Fisher Scientific), 1% penicillin/streptomycin cocktail (Gibco^TM^; Thermo Fisher Scientific) and 0.1% amphotericin B (Gibco^TM^; Thermo Fisher Scientific). All the homogenous sub-cultures of mitotically stable PEK lines were maintained in CO_2_ Incubator (Galaxy 170R, New Brunswick, NJ, USA) for 7 days at 37 °C in a 100% water-saturated atmosphere of 5% CO_2_ and 95% air. Medium intended for the ex vivo expanding the PEKs was replenished every 3 days. For immunofluorescence and lectin staining, the cells were plated into 8-well microscopic chamber slides (LabTek^TM^ CC2 Nunc; Thermo Fisher Scientific) and then perpetuated under such proliferative and adherent subculture conditions until they reach a subconfluence at the level of approximately 85%.

### 4.3. Immunofluorescence Dyeing of the Ex Vivo-Expanded Single-, Double- and Nontransgenic PEKs

The PEKs were washed with ice-cold PBS (Biomed) and fixed with cold 4% paraformaldehyde (PFA; Sigma–Aldrich) in PBS for 10 min. After several washes in PBS (Biomed), they were blocked in 5% normal goat serum in PBST (phosphate-buffered saline with the addition of 0.1% *v*/*v* Triton X-100; Bioshop Inc., Burlington, VT, Canada) for 30 min. The PEKs were subsequently incubated overnight at +4 °C in a humidified chamber with the appropriate primary antibodies (i.e., rabbit polyclonal immunoglobulins isotype G; IgGs) against: human α1,2-FT (diluted 1:150 in PBST; ab198712, Abcam, Cambridge, UK) and human α-Gal A (diluted 1:200 in PBST; PA5-27349, Thermo Fisher Scientific, Waltham, MA, USA). Afterwards, the cells were rinsed repeatedly in PBST followed by 1-h exposure to either goat anti-rabbit Cy3-tagged secondary antibodies, i.e., IgGs against human α1,2-FT (diluted 1:600 in PBST; Jackson ImmunoResearch Laboratories, Inc., West Grove, Chester County, PA, USA) or goat anti-rabbit Alexa Fluor 488-tagged secondary antibodies, i.e., IgGs against human α-Gal A (diluted 1:600 in PBST; Thermo Fisher Scientific) at room temperature. In the next step, the cell sections that were previously washed several times were counterstained with 4′,6-diamidino-2-phenylindole (DAPI) in Fluoroshield Antifade Mounting Medium (F6057, Sigma-Aldrich), whose unique formula enables to retain prolonged fluorescence not only by preventing rapid photobleaching (fading) of Cy3 and Alexa Fluor 488 fluorochromes, but also by minimizing the blinking-mediated phenomena resulting from extended lifetime and long-term excitation of previously mentioned fluorescent dyes. Finally, fluorescently tagged cells were mounted onto precleaned glass microscope slides under coverslipes, and then evaluated as was described in paragraph “*4.5. Confocal Microscope Analyses of the Ex Vivo-Expanded Single-, Double- and Nontransgenic PEKs*”.

### 4.4. Lectin-Mediated Labelling of Galα1→3Gal Epitopes in the Ex Vivo-Expanded Single-, Double- and Nontransgenic PEKs

The localization of Galα1→3Gal antigenic determinants and subsequent comparative assessment of their semiquantitative expression profiles (RAs) in the PEKs from both CTR nTG group and each transgenic variant (h*FUT2*, h*GLA* and h*FUT2*×h*GLA*) were accomplished by using Alexa Fluor 647 fluorochrome-conjugated lectin/isolectin GS I-B_4_ displaying a strict specificity and strong affinity for terminal αGal residues. The isolectin GS I-B_4_ is a member representing the family of tetrameric phytohemagglutinins that were isolated from the seeds of the tropical African legume shrub *Griffonia simplicifolia* (I32450, Molecular Probes, Invitrogen^TM^, Thermo Fisher Scientific). The PEKs were rinsed with ice-cold PBS (Biomed) and then fixed with cold 4% PFA (Sigma-Aldrich) in PBS for 10 min. Following serial PBS-mediated washes, they were blocked in 1% bovine serum albumin (BSA; Bioshop Inc., Burlington, VT, Canada) in PBST for 1 h. After repeatedly rinsing with PBS, the PEKs underwent the overnight exposure to isolectin GS I-B_4_ (diluted 1:200 in DPBS) at +4 °C in a dark humidified chamber. Finally, cells were washed thrice in PBS followed by mounting in DAPI-supplemented Fluoroshield Medium (comprised of antifade reagent with antiphotobleaching and anti-blinking properties that allow for perpetuating and extending the lifetime of Alexa Fluor 647 fluorochrome-derived fluorescent signals). After the fluorescently tagged PEKs of various genetically engineered models (h*FUT2*, h*GLA* and h*FUT2*×h*GLA*) and their nontransgenic counterparts were successfully coverslipped, they were subjected to comparative estimation of RAs noticed for Galα1→3Gal epitopes according to the protocol thoroughly described in paragraph “*4.5. Confocal Microscope Analyses of the Ex Vivo-Expanded Single-, Double- and Nontransgenic PEKs*”.

### 4.5. Confocal Microscope Analyses of the Ex Vivo-Expanded Single-, Double- and Nontransgenic PEKs

Fluorescently labelled cells were assessed by using laser scanning confocal microscope Olympus FluoView 1200 on inverted stand IX83 (Olympus, Tokyo, Japan). Forty-times magnification objective (NA = 0.95) was used, and diode laser (473 nm), diode laser (543 nm), diode laser (635 nm) and diode laser (405 nm) were applied to excite green (Alexa Fluor 488), red (Cy3), far-red (Alexa Fluor 647) and blue (DAPI) fluorescence, respectively. Relative intensities of fluorescence were quantified in each, randomly chosen region of interest (ROI) using ImageJ version 1.46r software (National Institutes of Health, Bethesda, MD, USA) in a greyscale of 256 levels [38,39]. Both for each variant of genetically modified PEKs (h*FUT2*, h*GLA* and h*FUT2*×h*GLA*) and for CTR nTG PEKs, three sections were sampled by 70 ROI.

### 4.6. Total Protein Extraction and Western or Lectin Blot Analyses Accomplished to Determine RAs Estimated for rhα1,2-FT and rhα-Gal A Enzymes or Galα1→3Gal Epitopes at the Protein Levels in the Ex Vivo-Expanded Single-, Double- and Nontransgenic PEKs

Total protein was extracted from the in vitro proliferating and detached PEKs of all the transgenic types/models (h*FUT2*, h*GLA* and h*FUT2*×h*GLA*) and their nontransgenic cell counterparts by using radioimmunoprecipitation assay lysis buffer (RIPA buffer; Thermo Fisher Scientific) containing 1% of proteinase inhibitor cocktail (RIPA+PI; Bioshop Inc., Burlington, VT, Canada). Following the first passage, the PEKs were cultured in T-25 flasks for 7 days to reach a total confluence. Afterwards, the cells were rinsed twice in ice-cold PBS (Biomed) and then 300 µL of RIPA+PI was added per single culture flask, followed by harvesting the PEKs with the use of cell scrapers. In the next step, cell lysates were sonicated and centrifuged at 14,000× *g* for 15 min at +4 °C, followed by collection of supernatants. Protein concentration was determined with the aid of microassay DC^TM^ Protein Assay (Bio-Rad Laboratories, Hercules, CA, USA) using BSA (Sigma–Aldrich) as a standard. Protein samples were stored at –80 °C for subsequent analyses.

For sodium dodecyl-sulphate (SDS)-polyacrylamide gel electrophoresis (SDS-PAGE), protein samples were diluted in 2× Laemmli Sample Buffer (Bio-Rad Laboratories) containing 5% β-mercaptoethanol and denatured at 100 °C per 5 min. Then, the proteins were separated in SDS-PAGE using 5% stacking and 10% resolving gels. Molecular weights of the analyzed proteins were estimated with reference to standard proteins (Precision Plus Dual Color Protein Standard; Bio-Rad Laboratories). For immunoblotting and lectin blotting, proteins were electro-transferred onto a poly(vinylidene fluoride) (PVDF) membrane (Immobilon-P; Merck, Darmstadt, Germany) at constant amperage of 250 mA for 120 min.

Taking into account the immunoblotting, membranes were blocked for 1 h in 5% non-fat milk in TBST (Tris-buffered saline with 0.1% *v*/*v* Tween20; Bioshop Inc.) and, after several rinses with TBST, incubated overnight at +4 °C with the following primary antibodies (the same as those for immunofluorescent labelling): rabbit polyclonal IgGs against human α1,2-FT (diluted 1:1000 in TBST; ab198712, Abcam) and rabbit polyclonal IgGs against human α-Gal A (diluted 1:1000 in TBST; PA5-27349, Thermo Fisher Scientific). β-Actin was used as a reference protein (mouse monoclonal IgG designated as anti-β-actin, diluted 1:2000 in TBST; ab8224, Abcam). Afterwards, membranes were washed several times in TBST followed by 1-h incubation with appropriate HRP-conjugated secondary antibodies (i.e., goat anti-rabbit IgGs against human α1,2-FT and goat anti-mouse IgGs against β-actin; Thermo Fisher Scientific), each at a dilution of 1:6000 in TBST, at room temperature [40].

Taking into consideration the lectin blotting, membranes were blocked for 30 min in 1% BSA in TBST (Bioshop Inc.). After several washes in Ca^2+^- and Mg^2+^-enriched Dulbecco’s phosphate-buffered saline (DPBS; Gibco, Thermo Fisher Scientific), followed by rinsing with TBS (Bioshop Inc.), membranes were subjected to overnight incubation with HRP-labelled isolectin GS I-B_4_ (L5391; Sigma-Aldrich) at a dilution of 1:2000 in DPBS and, then, underwent a terminal washing in TBS.

Finally, all the protein bands were detected by chemiluminescence using Clarity^TM^ Western ECL Blotting Substrate (Bio-Rad Laboratories) and visualized with the ChemiDoc^TM^ XRS+ Imaging System (Bio-Rad Laboratories). Protein bands were semiquantified using the Image Lab^TM^ 2.0 Software (Bio-Rad Laboratories) by measurement of their relative optical densities (RODs). Following detection of the bands related to the analyzed protein samples, membranes were stripped and reprobed with anti-β-actin antibody for loading control (i.e., reference) protein.

### 4.7. Statistical Analysis

For each variant/model of genetically engineered and nonengineered PEKs and for all the analyses carried out, three repeats were performed. Semiquantitative data were expressed in the form of the mean ± standard error of the mean (SEM) and subjected to statistical estimation by using the Shapiro–Wilks *W* test for normality to determine whether a variable that is presumed to cause a change in another variable is normally distributed in a population. The accomplishments of comparatively analyzing between the assigned means ± SEMs were mediated by one-way analysis of variance (ANOVA) and subsequent Tukey’s honestly significant difference (HSD) post hoc test. The incidences of statistically significant intergroup variability with the probabilities of occurring random errors at the levels of *p* < 0.05, *p* < 0.01, and *p* <0.001 were denoted as follows: single superscript asterisks (*), double superscript asterisks (**), and triple superscript asterisks (***), respectively.

## 5. Conclusions

The efforts targeted at establishing and cryogenically protecting stable and homogenous cell lines of single- and double-transgenic PEKs that are characterized by not only ameliorated expression levels of rhα-1,2-FT and/or rhα-Gal A proteins, but also lessened/attenuated semiquantitative profiles of Galα1→3Gal epitopes were efficiently undertaken for the first time. To the best of our knowledge, the current investigation is also the first to thoroughly and simultaneously specify the RAs noticed for extrinsic rhα1,2-FT and rhα-Gal A immune-related enzymes followed by augmented decline in the levels of Galα1→3Gal antigenic determinants among the ex vivo-expanded PEK lines that were successfully generated and clonally multiplied from cutaneous bioptates stemming from h*FUT2*×h*GLA* bi-transgenic pigs.

The aforementioned strategies will enable us to carry out further studies focused on cloning the genetically engineered pigs by SCNT for the needs of clinical research designed to develop and optimize the negligibly intrusive and nontraumatic procedures of regenerative medicine and reconstructive surgery of human dermo-integumentary system. The latter encompass dermoplasty-based therapies aimed either at repairing the injuries in human cutaneous and subcutaneous tissues or at minimizing and eliminating the impairments in human skin integrity by porcine mono- and/or bi-genetically modified dermo-epidermal xenotransplants. Collectively, the concept of using single- or double-transgenic epidermal keratinocytes as a source of adult donor cells for SCNT represents an entirely novel approach both in pigs and in other mammalian species.

## Data Availability

Not applicable.

## Abbreviations

HAR	Hyperacute rejection
HRP	Horseradish peroxidase
LacNAc	*N*-Acetyllactosamine
NDECs	Nuclear donor epidermal cells
rhα1,2-FT	Recombinant human α1,2-fucosyltransferase
rhα-Gal A	Recombinant human α-galactosidase A
α1,3-GT	α1,3-Galactosyltransferase
PEKs	Porcine epidermal keratinocytes
RA	Relative abundance
SCNT	Somatic cell nuclear transfer
UDP-Gal	Uridine 5′-diphosphogalactose; Galactose-uridine-5′-diphosphate; UDP-α-D-galactose

## References

[1] Niemann H.P. (2016). The production of multi-transgenic pigs: Update and perspectives for xenotransplantation. Transgenic Res..

[2] Cooper D.K., Gollackner B., Sachs D.H. (2002). Will the pig solve the transplantation backlog?. Annu. Rev. Med..

[3] Galili U., Shohet S.B., Kobrin E., Stults C.L., Macher B.A. (1988). Man, apes, and Old World monkeys differ from other mammals in the expression of alpha-galactosyl epitopes on nucleated cells. J. Biol. Chem..

[4] Hryhorowicz M., Zeyland J., Słomski R., Lipiński D. (2017). Genetically modified pigs as organ donors for xenotransplantation. Mol. Biotechnol..

[5] Whyte J.J., Prather R.S. (2011). Genetic modifications of pigs for medicine and agriculture. Mol. Reprod. Dev..

[6] Cooper D.K.C., Ekser B., Tector A.J. (2015). Immunobiological barriers to xenotransplantation. Int. J. Surg..

[7] Cooper D.K., Dou K.F., Tao K.S., Yang Z.X., Tector A.J., Ekser B. (2016). Pig liver xenotransplantation: A review of progress toward the clinic. Transplantation.

[8] Lu T., Yang B., Wang R., Qin C. (2020). Xenotransplantation: Current status in preclinical research. Front. Immunol..

[9] Sandrin M.S., McKenzie I.F. (1994). Gal alpha (1,3)Gal, the major xenoantigen(s) recognised in pigs by human natural antibodies. Immunol. Rev..

[10] Blanken W.M., van den Eijnden D.H. (1985). Biosynthesis of terminal Gal α1→3Galβ1→4GlcNAc-R oligosaccharide sequences on glycoconjugates. Purification and acceptor specificity of a UDP-Gal: *N*-acetyllactosaminide α1→3-galactosyltransferase from calf thymus. J. Biol. Chem..

[11] Osman N., McKenzie I.F., Ostenried K., Ioannou Y.A., Desnick R.J., Sandrin M.S. (1997). Combined transgenic expression of alpha-galactosidase and alpha1,2-fucosyltransferase leads to optimal reduction in the major xenoepitope Galalpha(1,3)Gal. Proc. Natl. Acad. Sci. USA.

[12] Varki A. (1998). Factors controlling the glycosylation potential of the Golgi apparatus. Trends Cell Biol..

[13] Hartel-Schenk S., Minnifield N., Reutter W., Hanski C., Bauer C., Morré D.J. (1991). Distribution of glycosyltransferases among Golgi apparatus subfractions from liver and hepatomas of the rat. Biochim. Biophys. Acta.

[14] Luo Y., Wen J., Luo C., Cummings R.D., Cooper D.K.C. (1999). Pig xenogeneic antigen modification with green coffee bean α-galactosidase. Xenotransplantation.

[15] Zeyland J., Woźniak A., Gawrońska B., Juzwa W., Jura J., Nowak A., Słomski R., Smorąg Z., Szalata M., Mazurek U. (2014). Double transgenic pigs with combined expression of human α1,2-fucosyltransferase and α-galactosidase designed to avoid hyperacute xenograft rejection. Arch. Immunol. Ther. Exp..

[16] Li J., Greco V., Guasch G., Fuchs E., Mombaerts P. (2007). Mice cloned from skin cells. Proc. Natl. Acad. Sci. USA.

[17] Izumi K., Feinberg S.E. (2002). Skin and oral mucosal substitutes. Oral Maxillofac. Surg. Clin. N. Am..

[18] Izumi K., Takacs G., Terashi H., Feinberg S.E. (1999). *Ex vivo* development of a composite human oral mucosal equivalent. J. Oral Maxillofac. Surg..

[19] Wanichpakorn S., Kedjarune-Laggat U. (2010). Primary cell culture from human oral tissue: Gingival keratinocytes, gingival fibroblasts and periodontal ligament fibroblasts. Songklanakarin J. Sci. Technol..

[20] Xu H., Wan H., Sandor M., Qi S., Ervin F., Harper J.R., Silverman R.P., McQuillan D.J. (2008). Host response to human acellular dermal matrix transplantation in a primate model of abdominal wall repair. Tissue Eng. Part A.

[21] Wiater J., Karasiński J., Słomski R., Smorąg Z., Wartalski K., Gajda B., Jura J., Romek M. (2020). The effect of recombinant human alpha-1,2-fucosyltransferase and alpha-galactosidase A on the reduction of alpha-gal expression in the liver of transgenic pigs. Folia Biol..

[22] Lipinski D., Jura J., Zeyland J., Juzwa W., Mały E., Kalak R., Bochenek M., Plawski A., Szalata M., Smorag Z. (2010). Production of transgenic pigs expressing human a1,2-fucosyltransferase to avoid humoral xenograft rejection. Med. Weter..

[23] Ramirez P., Montoya M.J., Ríos A., García Palenciano C., Majado M., Chávez R., Muñoz A., Fernández O.M., Sánchez A., Segura B. (2005). Prevention of hyperacute rejection in a model of orthotopic liver xenotransplantation from pig to baboon using polytransgenic pig livers (CD55, CD59, and H-transferase). Transplant. Proc..

[24] Lutz A.J., Li P., Estrada J.L., Sidner R.A., Chihara R.K., Downey S.M., Burlak C., Wang Z.Y., Reyes L.M., Ivary B. (2013). Double knockout pigs deficient in N-glycolylneuraminic acid and Galactose α-1,3-Galactose reduce the humoral barrier to xenotransplantation. Xenotransplantation.

[25] Sahara H., Watanabe H., Pomposelli T., Yamada K. (2017). Lung xenotransplantation. Curr. Opin. Organ Transplant..

[26] Klymiuk N., Aigner B., Brem G., Wolf E. (2010). Genetic modification of pigs as organ donors for xenotransplantation. Mol. Reprod. Dev..

[27] Bottino R., Wijkstrom M., van der Windt D.J., Hara H., Ezzelarab M., Murase N., Bertera S., He J., Phelps C., Ayares D. (2014). Pig-to-monkey islet xenotransplantation using multi-transgenic pigs. Am. J. Transplant..

[28] Kong S., Ruan J., Xin L., Fan J., Xia J., Liu Z., Mu Y., Yang S., Li K. (2016). Multi-transgenic minipig models exhibiting potential for hepatic insulin resistance and pancreatic apoptosis. Mol. Med. Rep..

[29] Kwon D.J., Kim D.H., Hwang I.S., Kim D.E., Kim H.J., Kim J.S., Lee K., Im G.S., Lee J.W., Hwang S. (2017). Generation of α-1,3-galactosyltransferase knocked-out transgenic cloned pigs with knocked-in five human genes. Transgenic Res..

[30] Fischer K., Kraner-Scheiber S., Petersen B., Rieblinger B., Buermann A., Flisikowska T., Flisikowski K., Christan S., Edlinger M., Baars W. (2016). Efficient production of multi-modified pigs for xenotransplantation by ‘combineering’, gene stacking and gene editing. Sci. Rep..

[31] Zeyland J., Gawrońska B., Juzwa W., Jura J., Nowak A., Słomski R., Smorąg Z., Szalata M., Woźniak A., Lipiński D. (2013). Transgenic pigs designed to express human α-galactosidase to avoid humoral xenograft rejection. J. Appl. Genet..

[32] Haruyama N., Cho A., Kulkarni A.B. (2009). Overview: Engineering transgenic constructs and mice. Curr. Protoc. Cell Biol..

[33] Kong Q., Wu M., Huan Y., Zhang L., Liu H., Bou G., Luo Y., Mu Y., Liu Z. (2009). Transgene expression is associated with copy number and cytomegalovirus promoter methylation in transgenic pigs. PLoS ONE.

[34] Houdebine L.M. (2005). Use of transgenic animals to improve human health and animal production. Reprod. Domest. Anim..

[35] Clark A.J., Bissinger P., Bullock D.W., Damak S., Wallace R., Whitelaw C.B.A., Yull F. (1994). Chromosomal position effects and the modulation of transgene expression. Reprod. Fertil. Dev..

[36] Garrick D., Fiering S., Martin D.I.K., Whitelaw E. (1998). Repeat-induced gene silencing in mammals. Nat. Genet..

[37] Folger K.R., Wong E.A., Wahl G., Capecchi M.R. (1982). Patterns of integration of DNA microinjected into cultured mammalian cells: Evidence for homologous recombination between injected plasmid DNA molecules. Mol. Cell. Biol..

[38] Gajda B., Romek M., Grad I., Krzysztofowicz E., Bryla M., Smorag Z. (2011). Lipid content and cryotolerance of porcine embryos cultured with phenazine ethosulfate. Cryo Lett..

[39] Romek M., Gajda B., Krzysztofowicz E., Kucia M., Uzarowska A., Smorag Z. (2017). Improved quality of porcine embryos cultured with hyaluronan due to the modification of the mitochondrial membrane potential and reactive oxygen species level. Theriogenology.

[40] Wiater J., Samiec M., Skrzyszowska M., Lipiński D. (2021). Trichostatin A-Assisted Epigenomic Modulation Affects the Expression Profiles of not only Recombinant Human α1,2-Fucosyltransferase and α-Galactosidase a Enzymes but also Galα1→3Gal Epitopes in Porcine Bi-Transgenic Adult Cutaneous Fibroblast Cells. Int. J. Mol. Sci..

